# How do healthcare professionals respond to ethical challenges regarding information management? A review of empirical studies

**DOI:** 10.1080/11287462.2021.1909820

**Published:** 2021-04-05

**Authors:** Cornelius Ewuoso, Susan Hall, Kris Dierickx

**Affiliations:** a Department of Medicine, University of Cape Town, South Africa; b Center for Applied Ethics, Stellenbosch University, Western-Cape, South Africa; c Centre for Biomedical Ethics and Law, Katholieke Universiteit Leuven, Leuven, Belgium

**Keywords:** Ethical challenges, information management, clinical context, systematic review, empirical literature

## Abstract

**Aim:**

This study is a systematic review that aims to assess how healthcare professionals manage ethical challenges regarding information within the clinical context.

**Method and Materials:**

We carried out searches in PubMed, *Google Scholar* and Embase, using two search strings; searches generated 665 hits. After screening, 47 articles relevant to the study aim were selected for review. Seven articles were identified through snowballing, and 18 others were included following a system update in PubMed, bringing the total number of articles reviewed to 72. We used a Q-sort technique for the analysis of identified articles.

**Findings:**

This study reveals that healthcare professionals around the world generally employ (to varying degrees) four broad strategies to manage different types of challenges regarding information, which can be categorized as challenges related to confidentiality, communication, professional duty, and decision-making. The strategies employed for managing these challenges include resolution, consultation, stalling, and disclosure/concealment.

**Conclusion:**

There are a variety of strategies which health professionals can adopt to address challenges regarding information management within the clinical context. This insight complements current efforts aimed at enhancing health professional-patient communication. Very few studies have researched the results of employing these various strategies. Future empirical studies are required to address this.

**Abbreviations:**

CIOMS: Council of International Organization of Medical Sciences; WHO: World Health Organization; AMA: American Medical Association; WMA: World Medical Association; PRISMA: Preferred Reporting Items for Systematic Reviews and Meta-Analysis; ISCO: International Standard Classification of Occupations; ILO: International Labour Office; SPSS: The Statistical Package for the Social Sciences

## Background

Information management in the clinical setting is essential to good patient care. Poor management of information can negatively affect the health professional-patient relationship or jeopardize patient health. However, it is not always clear how information should be managed within the clinical context. Information management here is understood as the power of health professionals to control the disclosure or withholding of information to their patients (Ewuoso et al., 2017). This encompasses information about diagnosis, prognosis, and preferred available therapy (Swaminath, 2008).

Current inter/national regulations and professional bodies^1^ generally require health professionals such as physicians to disclose information with significant welfare implications, whether health-related or psychological, fully and accurately to their patients. Such disclosure, these bodies hold, would significantly strengthen patient autonomy and enhance informed decision-making. This obligation is a matter of ethics and law. Withholding relevant information from patients that may guide them in making decisions about what course of treatment to pursue, represents a violation of the patient’s right to informed decision-making (Ewuoso et al., 2017).

A failure to disclose information could also expose a health professional to legal liability (Murray, 2012). However, regulations such as The Healthcare Professions Council of South Africa’s Guidelines for Good Practice in the Healthcare Professions (2008, Booklet 4), permit a health professional to withhold information in circumstances where disclosure is medically contraindicated^2^ for example, where disclosure may lead to harm or compromise the patient’s recovery process.

However, there are some clinical encounters where deciding what course of action to take with respect to the management of information may prove extremely difficult. This is the situation when a health professional encounters a genuine moral dilemma, such as when incidental information with significant implications is accidentally discovered within the clinical context (for example, where misattributed paternity is discovered in the course of genetic testing). Disclosing such incidental information, where establishing such information is not the purpose of the test conducted, could be taken as a breach of one’s right “not to know”, as discussed, for example, by Andorno (2004) and Laurie (2014). Non-disclosure, on the other hand, could also be taken as a violation of one’s right to know. Beauchamp and Childress (2009, p. 10f) define a genuine moral dilemma as a puzzling circumstance “in which moral obligations demand or appear to demand that a person adopt each of two (or more) alternative but incompatible actions, such that the person cannot perform all the required actions.” In other words, a genuine dilemma is a situation in which an individual competently judges that they are morally obligated to perform A, but cannot due to other compelling arguments mandating “not A”. Other contingent circumstances also negate performing A and not A at the same time (Ewuoso et al., 2017).

This project sets out to assess the management of ethical challenges regarding information by health professionals. Specifically, this project is concerned with the question: how do healthcare professionals respond to ethical challenges regarding information management that arise in the clinical context? There is a large body of empirical literature that has attempted to answer this question in various ways. This study attempts to synthesize these findings in the form of a systematic review, carefully highlighting the broad types of challenges that health professionals face in this regard. Additionally, this study aims to provide insight into the various approaches/strategies used by professionals to deal with these challenges, as well as the moral framework(s) underlying those approaches/strategies. In the next section, the study describes its methodology for retrieving and including relevant literature.

## Method and Materials

### Literature Search

This study will adopt the Preferred Reporting Items for Systematic Reviews and Meta-Analysis (PRISMA) framework. The quality assessment for each study will be based on Appendix C of Hawker and colleagues’ (Hawker et al., 2002) *Appraising the evidence: reviewing disparate data systematically.* This assessment tool comprises of nine questions, each of which can be answered with “good”, “fair”, “poor”, and “very poor”. These ratings were converted into numerical figures: good (7-10); fair (5-6); poor (2-4); and very poor (1). The numbers were summed up to create an overall quality grade: 70–90 being high-quality grade, 50–69 medium quality, and below 50 low-quality grade.

Two searches were conducted in *PubMed* on the 27th of October and 28th of October 2016, to identify empirical studies that focus on the management of ethical challenges regarding information by health professionals in the clinical context. The term “health professional” is defined by the International Standard Classification of Occupations (ISCO, 2012) as generalists and special practitioners, pharmacists, nurses, and dentists. We developed our search strings for retrieving relevant literature by adopting the approach established by Pillastrini et al. (2015). This approach consists of (1) framing a research question, (2) looking up Medical Subject Heading (MeSH) terms for the components which make up the research question, (3) reading published literature for alternative terms and finally, (4) combining these MeSH terms with Boolean operators (AND, OR, and NOT) in a database to deliver relevant articles. These search strings generated several hits. A detailed description of these is provided in Table 1.

**Table 1.** Search for literature.

Literature Search
Search Date: 19 May 2020 Selected Restrictions: no restriction selected Search Mode: Default mode: sort by relevance Search String: ((((dilemma AND information)) AND (ethic OR ethics OR ethical OR moral)) AND (disclosure OR concealment OR “non disclosure” OR reporting OR returning OR “truth telling” OR recontacting OR withholding OR communicat*)) AND (intern OR surgeon OR nurse OR Allied Health Personnel OR caregiver OR dentist OR pharmacist OR geneticist OR “genetic counselor” OR oncologist OR physician OR “general practitioner” OR “foreign medical graduate” OR resident OR anatomist OR psychiatrist OR “clinical scientist” OR GP) Additional Documents: 12
Search Date: 13 January 2018 Selected Restrictions: no restriction selected Search Mode: Default mode: sort by relevance Search String: ((((dilemma AND information)) AND (ethic OR ethics OR ethical OR moral)) AND (disclosure OR concealment OR “non disclosure” OR reporting OR returning OR “truth telling” OR recontacting OR withholding OR communicat*)) AND (intern OR surgeon OR nurse OR Allied Health Personnel OR caregiver OR dentist OR pharmacist OR geneticist OR “genetic counselor” OR oncologist OR physician OR “general practitioner” OR “foreign medical graduate” OR resident OR anatomist OR psychiatrist OR “clinical scientist” OR GP) Additional: 6
Search Date: 27 October 2016 Selected Restrictions: no restriction selected Search Mode: Default mode: sort by relevance Search String: ((((dilemma AND information)) AND (ethic OR ethics OR ethical OR moral)) AND (disclosure OR concealment OR “non disclosure” OR reporting OR returning OR “truth telling” OR recontacting OR withholding OR communicat*)) AND (intern OR surgeon OR nurse OR Allied Health Personnel OR caregiver OR dentist OR pharmacist OR geneticist OR “genetic counselor” OR oncologist OR physician OR “general practitioner” OR “foreign medical graduate” OR resident OR anatomist OR psychiatrist OR “clinical scientist” OR GP) Hits: 114
Data base: PubMed Choose search number: Second Search
Search Date: 28 October 2016 Selected Restrictions: no restriction selected Search Mode: Default mode: sort by relevance Search String: (((((ethic OR ethics OR ethical OR moral)) AND (clinic or clinical)) AND (dilemma OR complex* OR conflict)) AND (disclosure OR concealment OR “non disclosure” OR reporting OR returning OR recontacting OR withholding OR communicat*)) AND (intern OR surgeon OR nurse OR Allied Health Personnel OR caregiver OR dentist OR pharmacist OR geneticist OR “genetic counselor” OR oncologist OR physician OR “general practitioner” OR “foreign medical graduate” OR resident OR anatomist OR psychiatrist) Hits: 306
Data base: Google Scholar Choose search number: Additional search
Search Date: 28 October 2016 Selected Restrictions: no restriction selected Search Mode: Default mode: sort by relevance Search String: (((((ethic OR ethics OR ethical OR moral)) AND (clinic or clinical)) AND (dilemma OR complex* OR conflict)) AND (disclosure OR concealment OR “non disclosure” OR reporting OR returning OR recontacting OR withholding OR communicat*)) AND (intern OR surgeon OR nurse OR Allied Health Personnel OR caregiver OR dentist OR pharmacist OR geneticist OR “genetic counselor” OR oncologist OR physician OR “general practitioner” OR “foreign medical graduate” OR resident OR anatomist OR psychiatrist) Hits: 210
Data base: Embase Choose search number: Additional search
Search Date: 15 November 2016 Selected Restrictions: No restriction selected Search Mode: None selected Key phrase: (((((ethic OR ethics OR ethical OR moral)) AND (clinic or clinical)) AND (dilemma OR complex* OR conflict)) AND (disclosure OR concealment OR “non disclosure” OR reporting OR returning OR recontacting OR withholding OR communicat*)) AND (intern OR surgeon OR nurse OR Allied Health Personnel OR caregiver OR dentist OR pharmacist OR geneticist OR “genetic counselor” OR oncologist OR physician OR “general practitioner” OR “foreign medical graduate” OR resident OR anatomist OR psychiatrist) Hits: 35

Table 1 shows a detailed description of the literature search; that, is search date, search string, search number, filters applied and hits generated.

The study conducted additional searches using a broad search string in *Google Scholar* (generating 210 hits) and Embase (generating 35 hits). After screenings for language (22 articles excluded), title and abstract (535 articles excluded), year of publication (only empirical articles published between 2000 and 2016 included – 28 articles excluded), duplicates (10 articles excluded), and full text (12 articles excluded), 47 articles were included. Seven articles were identified through snowballing. Six other articles were identified on January 13, 2018, and 12 more articles on the 19th of May 2020, following a system update in PubMed. 72 articles were eventually included in this systematic review (See Table 2 above).

**Table 2.** Selection process.

Table 2 shows how exclusion and inclusion criteria, such as language, date of publication, full-text screenings, etc., were applied to identify articles for review.

### Inclusion and Exclusion Criteria

This systematic review focuses only on empirical studies^3^ that, (1) aim to address how health professionals (as defined by ISCO) manage dilemmas or challenges regarding information in the clinical context, (2) identify factors influencing the choice of strategy by healthcare professionals for dealing with such challenges, and (3) study various forms of ethical challenges regarding information experienced by healthcare professionals. Non-empirical studies, such as concept-based articles, ethical guidelines, commentaries, case analyses, opinion papers, editorials, panel discussions, summary reports, letters, argument-based studies, or theoretical studies relating to ethical challenges in information management were excluded from this review.

The first author was responsible for the selection of articles for review, but this was discussed extensively with the co-authors to ensure consistency with regard to inclusion and exclusion criteria. Articles that met our selection criteria were pooled together in the EndNote database (version X6; Thomson Reuters).

### Data Extraction

Articles that met our inclusion criteria were conventionally analyzed using Q-sort methodology to extract data. Q-sort technique is a useful technique for qualitatively sorting large volumes of varying opinions into broader groups, by identifying common themes or highlighting how viewpoints are interconnected or related. As Watts and Stenner (2005, p. 74f) explain, Q-sort is mainly an exploratory methodology. The goal of this approach is to bring a sense of coherence to individual viewpoints or research questions that have many potentially complex and socially contested answers (Ewuoso et al., 2017; Roberts et al., 2015 Watts & Stenner, 2005;). Q-sort technique also has vertical and horizontal methodological usefulness. It involves a vertical progression from a well-defined research question to method, result, and discussion. Horizontally, this technique proceeds by coding relevant studies and sorting them into themes or groups. Given this two-fold usefulness, this technique is a relevant technique for understanding the broad range of ethical issues regarding information, as well as how health professionals manage such challenges, within the clinical context.

Using this methodological approach, the broad areas of challenges which health professionals experience with respect to information, strategies and approaches for managing these challanges, as well as the moral reasoning behind those approaches, were identified and categorized into meaningful themes in Atlas.ti. Other information such as author(s), title, country of origin, study aims, participant description, year of publication, and participants’ specialties, were extracted using a data extraction form (to ensure some level of standardization). The result of this endeavor is provided in the results section below.

## Results

In this section, we shall present the result of our review by highlighting the broad types of challenges health professionals experience within the clinical context, the broad strategies employed to address these challenges, and the moral reasoning behind these strategies.

### Challenges

The study identified four broad types of challenges regarding information which have been experienced by various health professionals in a variety of clinical contexts. They include: (1) confidentiality-related challenges, (2) decision-making related challenges, (3) communication-related challenges, and (4) professional duty-related challenges.

Each broad category comprises a number of ethical issues. The broad issues in communication-related challenges are: “how much to disclose”, “to whom to disclose”, “what to disclose”, and “when to disclose”. For example, when clinically significant (and hereditary) information is discovered, clinical geneticists (Akpinar & Ersoy, 2014; Alliman et al., 2009; Bower et al., 2002; Elger et al., 2015; Erde et al., 2006; Falk et al., 2003 Fennig et al., 2004; Lapid et al., 2009; Lisker & Carnevale, 2006; Williams et al., 2002) often face the difficulty of deciding how much of this information to disclose. Should individuals aged 50 years and over be informed of these results if the condition is not clinically actionable? Should patients aged 12 years and under be informed of adult-onset diseases? Is there a duty to warn at-risk relatives? Would warning third parties lead to a breach of their rights not to know? Communication challenges also arise in the health professional-patient context when prognosis is unclear (Jurchak et al., 2017), or when health professionals are unsure of the “right” thing to do. For example, Australian general practitioners (Pickles et al., 2016) reported that they felt unsure about what the “right” thing to do is when asymptomatic men ask about prostate cancer screening. Some health professionals express frustration regarding the lack of formal guidance to direct their practice, and many have found that talking with men about prostate-specific antigen (PSA) testing is a challenging experience because of this underlying uncertainty. This lack of formal guidance may be due to an absence of clinical ethics consultancy services in some clinical contexts. For example, a majority of emergency physicians interviewed by Joseph et al. (2019), claim that they have no institutional guidelines or education on how to access patients’ decision-making capacities. Similarly, a majority of medical oncologists in a study conducted by George & Demir Kureci (2020) claim that the most common issue for them was the absence of clinical ethics consultancy to guide them when they encounter ethical challenges (regarding information).

Three main sub-categories in confidentiality related challenges include: (1) informing patients about the limits of confidentiality, (2) disclosing patient health information to an insurance company, public authority or employer, and (3) deciding between breaching confidential patient information to benefit significant others or at-risk third parties, or maintaining patient confidentiality. Doctors practicing sports medicine (Malcolm & Scott, 2014), for example, report experiencing extreme difficulty in maintaining patient’s confidential health information due to the physical environment they operate in. Confidentiality-related challenges are also frequently encountered by Mental Health professionals (Akpinar & Ersoy, 2014; Alliman et al., 2009; Bower et al., 2002; Elger et al., 2015; Erde et al., 2006; Falk et al., 2003 Fennig et al., 2004; Lapid et al., 2009; Lisker & Carnevale, 2006; Lützén et al., 2000 Nash & Romanos, 2010; Vaga et al., 2016). One commonly reported ethical challenge in the preceding studies has to do with breaching confidential patient information to benefit a third party or to prevent harm to others. For example, deciding whether to ensure public safety by reporting a driver’s alcohol addiction without consent to their employer (a transport company), or to maintain the patient’s confidentiality, can be an ethical nightmare for these professionals.

The broad issues involved in professional duty-related challenges include conflicts between concealing emotions and fulfilling obligations towards patients. Yang et al. (2016), for example, have found that nurses are sometimes unable to express their personal beliefs regarding abortion. The delivery room routines and norms sometimes require nurses to participate in abortion, such as in cases of abortion due to non-chromosomal abnormalities. Other professional-duty related challenges include conflicts between the duty to report a colleague’s error and the desire to maintain their trust/friendship; and value conflicts, such as disagreements between the health professional and the patient or family members over the termination of pregnancy or pre-symptomatic testing of minors (Bower et al., 2002; Groepper et al., 2015). Honoring one’s duty to report abuse to the state can also be difficult for health professionals. For example, in Sweden (Kvist et al., 2014) where studies have linked child maltreatment or abuse to poor oral health, dentists are required to report any suspicion of child abuse in any child with poor oral health. Dentists (Kvist et al., 2014), however, express ethical difficulty in distinguishing a parent’s concern for a child’s wellbeing from child maltreatment; or child abuse from poor parenting.

In decision-making related challenges, the issues include disagreements within the medical team over treatment decisions (Huijer et al., 2000). For example, a majority of nurses in one study expressed the belief that an egalitarian model, in which nurses’ opinions are frequently sought, and in which patients and family members are not excluded from decision-making, is vital in enhancing communication within the clinical contexts. This belief sometimes brings nurses into conflict with physicians who believe themselves to be the experts, and upon whom others (including nurses) must depend (Molina-Mula et al., 2017). Communication difficulties amongst health professionals can also lead to poor communication with patients and family members. Even when information about treatment plans is communicated to patients and family members, disagreements can occur between professionals (who may, for example, consider a treatment plan to be in the best interest of the patients) and patients or family members (who may hold the view that the proposed treatment plan conflicts with their religious or cultural beliefs). Yoon et al. (2010) have found that conflicts or disagreements over treatment decisions are overlooked sources or signs of burnout among obstetricians and gynaecologists. These disagreements are also often reported to lead to treatment delays, as reported in some studies (Jurchak et al., 2017; Odeniyi et al., 2017; Span-Sluyter et al., 2018)

The four types of challenges identified above are described as occurring frequently by professionals in 17 different fields of practice within the clinical context, namely gynaecology/obstetrics, sports medicine, anaesthesiology, nursing, cardiology, oncology/palliative/intensive care medicine, family medicine, paediatrics, dentistry, general surgery, general practice, organ/tissue donation, mental healthcare, gerontology, laboratory genetics, clinical genetics, and among interns/medical students. The health professionals working in these areas describe their experience of these challenges in various ways. Paediatricians report that their experience of challenges regarding information is frustrating (Sørlie et al., 2000), surgeons in one study (Torjuul et al., 2005b) described their experience as stressful, tragic, and guilt-laden, while male nurses (Nordam et al., 2005) and mental health practitioners (Elger et al., 2015) complain that these challenges often lead to burnout. These emotions have been associated with moral distress by Prentice et al. (2016); Epstein and Hamric (2009); and Thomas and Mccullough (2015). Moral distress can also arise as a result of disagreements with family members. Lokker et al. (2018) have found that nurses experience moral distress in contexts in which they cannot act in a way which would, in their professional judgment, benefit their patients, because of disagreements (over treatment plans) with patients’ family members.

### Strategies

This review identified four broad strategies for managing the challenges discussed above. These include: (1) consultation (with colleagues, ethics committees or other professionals), (2) stalling (using delaying tactics such as using distraction to relax patients, continuing futile treatment, and pretending to have a plan), (3) resolution (having a prior discussion with patients or avoiding ethical dilemmas by referring patients to another hospital or health professional, seeking a court order or deferring decision-making to senior colleagues, and overriding patient’s autonomy), and (4) disclosure/concealment (honest disclosure, concealment, and lying).

Some oncologists, paediatrists, and other healthcare professionals in the intensive and palliative care units sometimes stall or consult with other colleagues or professionals (or ethics committees), when confronted with ethical challenges around withholding or continuing treatment, resource allocation, and other decision-making dilemmas (Morparia et al., 2012; Sørlie et al., 2000). A majority of nursing students maintain that most of the time, they defer to staff nurses when they experience micro-ethical dilemmas regarding patient autonomy or honoring best practices (Krautscheid & Brown, 2014). Staff nurses sometimes strive towards resolution by deferring to physicians when confronted with decision and communication-related challenges (Krautscheid & Brown, 2014; Van Zuuren & Van Manen, 2006; Watermeyer, 2015). Most mental healthcare practitioners, geneticists and clinicians address confidentiality-related challenges (Akpinar & Ersoy, 2014; Alliman et al., 2009; Elger et al., 2015; Erde et al., 2006; Falk et al., 2003; Groepper et al., 2015; Lützén et al., 2000), communication-related challenges (Erde et al., 2006; Swetz et al., 2007) and professional duty-related challenges (Bower et al., 2002; Lapid et al., 2009) through resolution (specifically, by having pre-discussions with patients or educating patients about advanced care planning before the occurrence of dilemmas), and consultation with family members and other colleagues or professionals. Registered nurses, physiotherapists, physicians and occupational therapists in studies conducted by Gronlund et al. (2016) and Velan (2019) expressed the belief that their professional experiences helped them to engage with patients and family members properly and handle ethical difficulties around value differences (which sometimes frustrate communication between health professionals and their patients). Lack of experience has been found by Kadivar et al. (2017) to negatively affect how paediatric residents address ethical dilemmas regarding information. A majority of dentists in a study conducted by Camoin et al. (2018) claimed that they sometimes sacrificed ethical values (such as patient autonomy) in order to provide beneficial care to anxious children with intellectual disabilities and to address decision-making related challenges; while Australian pharmacists (Hattingh & King, 2019) claim that they generally avoid situations they perceive as requiring complex management. Many Japanese physicians believe that few problems result when they honestly tell cancer patients about their poor prognosis (Elwyn et al., 2002).

Finally, our review shows that consultation is a widely used strategy by health professionals in oncology/palliative/end-of-life care, nursing, clinical genetics, laboratory genetics, paediatric, anaesthesiology, cardiology, dentistry, gynaecology, mental healthcare, general surgery, and among medical interns, to address challenges related to communication, professional duty, confidentiality, and decision-making.

### Moral Reasoning and Influencing Factors

Professionals justify the use of these strategies in a variety of ways. In a study conducted by Kagan et al. (2008), physicians and nurses said they would require colleagues with a confirmed diagnosis of a blood borne pathogen infection to disclose their medical situation to patients prior to surgery. These professionals claim that such infected colleagues should be restricted from performing invasive surgery since they constitute a danger to patients.^4^ Mental Health professionals in Elger et al. (2015) maintain that they would have a prior discussion with patients to inform them about the limits of confidentiality. Patients, these professionals maintain, have a right to know that confidentiality rules may not cover some information.

Furthermore, a majority of nurses in Helft and colleagues (Helft et al., 2011) said they could help patients prepare for the end of life by answering prognosis-related questions truthfully. Communicating in this way, these health professionals maintain, will allow patients to make informed decisions about their care. Similarly, Torjuul et al. (2005b) reported that surgeons who consulted with senior colleagues gained insights on how to handle incompetent colleagues or disagreement with patients or family members over treatment. Finally, Sørlie et al. (2000) reported that paediatricians sometimes stall when there is pressure to treat seriously ill new-born babies. That is, they pretend to have a solution or take their time. This, in their opinion, will give dying children and parents time to achieve death with dignity, and ensure fair distribution of resources.

In addition to the examples of moral reasoning identified above, there are other factors that health professionals claim sometimes influence their choice of strategy. For example, oncologists and professionals in intensive and palliative care units will also consider the age of patients, as well as patient comorbidity, the aggressiveness of the disease, and the effectiveness of treatment in their decision to withhold or continue treatment (Halvorsen et al., 2008 Lotz et al., 2016; Mccahill et al., 2002; Schimmer et al., 2012). Patients’ wishes and competencies are essential factors gastroenterologists and gynaecologists or obstetricians will consider in the decision to withhold or disclose information (Palmboom et al., 2007). Time pressure^5^, restricted space for privacy (owing to the role that family members play in treatment decisions^6^), reliance on traditional and religious treatments, and patient’s dependency on relatives for communication and decision-making regarding healthcare, are other factors (Kebede et al., 2020; Kwon et al., 2019; Wuensch et al., 2013) that sometimes cause delays in treatment (or preventive measures), or cause an unwillingness to discuss problems with health professionals openly.

Finally, 41 studies recommended additional training/education for health professionals. For example, donation physicians in one study (Macdonald & Shemie, 2017) believe that additional education on death determination (Chiu et al., 2009) for physicians and nurses, will minimize any risk of diagnostic errors, and enhance donation advocacy and donation conversion rates. Nineteen studies recommended new/additional guidelines and frameworks for managing challenges regarding information within the clinical context; 23 studies specifically recommended more ethics training that focuses on improving health professionals’ competence in different contexts (Beck et al., 2008; Cantini & Ells, 2007; Chih et al., 2016; Duval et al., 2004; Martin et al., 2014 Pye, 2013). Yildiz (2019), for example, recommended additional professional ethics training for nurses so that these professionals can better understand the ethical aspects of nursing. This will also enhance their competence in managing ethical dilemmas. Five studies strongly recommended that ethics education should focus on enhancing professionals’ competence in managing cultural/religious divides, which often exist between health professionals and patients. Cultural differences, as some studies (Buken & Balseven-Odabasi, 2013; Hurst et al., 2007 Malcolm & Scott, 2014; Ruhnke et al., 2000) have found, do indeed influence how doctors perceive or address ethical dilemmas. Health professionals require assistance in improving their practical competency in managing these differences.

## Discussion

This study provides insight into how healthcare professionals conceptualize the various challenges, with specific regard to information, which they experience within the clinical context, and the various strategies they employ for managing these challenges.

We observe, however, that our presentation of results in the previous section was weighted towards some of the 17 different fields of practice idenitified within the clinical context. It is not practically possible to discuss the ethical challenges experienced in each of the 17 fields of practice, as we lacked the space for such an enormous task. Our inability to discuss the ethical challenges in each of the 17 fields of practice may be taken as one limitation of this study. Regardless of this, we are optimistic that the insights gained through this study will significantly complement efforts aimed at enhancing healthcare professional-patient communication within the clinical context.

This discussion – as was the case with the presentation of findings – will be guided by our research question, which is: how do healthcare professionals respond to ethical challenges regarding information management that arise in the clinical context? This study identified challenges related to communication, decision-making, confidentiality, and professional duty as key themes for ethical reflection. It explored the experiences of healthcare professions in 17 different fields of practice within the clinical context. The study also identified four strategies – resolution, stalling, disclosure/concealment, and consultation – for managing these challenges.

Information management is an integral part of good patient care. The four broad types of challenges identified reveal the various ways in which communication^7^ amongst health professionals; or between health professionals and patients/families, could break down, or lead to the provision of information to a patient who is either unwilling or unprepared to receive such information, thus leading to avoidable harm or jeopardizing patient care. Hence, communication break-down, as well as the importance of forestalling this break-down, is the underlying ethical challenge regarding information experienced by professionals within the clinical context. The four broad strategies for managing ethical challenges around information show how professionals endeavor to prevent this harm from occurring, thereby improving professional-patient communication. Very few studies have considered the question as to whether these strategies lead to satisfactory outcomes in which all stakeholders – health professionals, patients, and family members – are made better-off. Further research is required to fill this gap.

The recommendation for new/additional frameworks or guidelines, as well as ethics education and training which focuses on enhancing health professional’s competence in clinical contexts (such as those contexts in which health professionals and patients are influenced by different religious/cultural beliefs), aligns with various calls (Godfrey et al., 2013; Westra et al., 2009) for theoretical diversity, as well as multiculturalism, in clinical ethics support systems and within the clinical context in general. For example, Westra and colleagues (Westra et al., 2009) have remarked that in non-religious ethics, the principle of non-maleficence may be used to justify withholding or withdrawing futile or damaging treatments or withholding damaging information. In contrast, Islamic ethics applies this principle to forbid all actions that may harm life. Similarly, Jegede (Jegede, 2009) has also expressed the concern that in some African contexts, autonomy would be understood as social autonomy, rather than individual self-determination, as is promoted by Beauchamp and Childress (2009). Additionally, ethics training in communication skills and ethical judgment will significantly improve health professionals’ ethical competence in managing such clinical situations. As Pettersson et al. (2018, p. 1) rightly observe,

ethical education and discussions for further development of a common ethical language and a good ethical working climate can improve ethical competence and help nurses and physicians cooperate better with regard to patients … in their efforts to act in the best interest of patients.

This additional training could also focus on enhancing health professionals’ multicultural competency. This is required to address social contexts, cultural milieu, religious beliefs, as well as other important values, which sometimes complicate how information is managed within the clinical context, or which could lead to communication break-down between health professionals and their patients. Additional ethics training can also complement the current medical ethics framework by focusing on providing grounds for justification of any loss of value that may occur in the event of ethical dilemmas. Given the current definition of genuine dilemmas (Beauchamp & Childress, 2009, p. 10f), it is unlikely that a loss of value can be prevented in the event of a real dilemma. As rightly observed by some of the reviewed studies (Cahana et al., 2008; Wuensch et al., 2013), in a real dilemma, some values or beliefs would be breached or suppressed in favor of others. What is needed – while not entirely foreclosing the possibility of developing a universal framework that embraces all beliefs and practices – is an adequate justification for such a violation or loss. Such justification will provide relief for healthcare professionals who experience these dilemmas and restore their confidence in a way that minimizes moral distress. Finally, in order for these additional ethical frameworks to have a lasting effect, this study recommends reform of healthcare guidelines/policies, and medical ethics education curricula to accommodate these new or additional theories.

In addition to the limitation already stated in this section, other limitations must also be noted. The eligibility criteria adopted by this study may have resulted in the exclusion of potentially relevant studies. For example, we included in this study only articles published between 2000 and 2018; this resulted in the exclusion of potentially relevant articles published before 2000. Notwithstanding the stated limitations, this study greatly complements efforts aimed at enhancing healthcare professional-patient communication, as well as clinical ethics support services. Future studies can build on the outcome of this study by focussing on developing training manuals and ethics education programs for enhancing health professional-patient communication.

## Conclusion

Insight has been gained, through this study, into how healthcare professionals respond to ethical dilemmas regarding information. This study complements efforts aimed at enhancing healthcare professional-patient communication. This review of empirical studies has identified challenges related to communication, decision-making, confidentiality, and professional duty as ethical issues that can affect healthcare professional-patient communication, in 17 different fields of practice within the clinical context. The study also identified four strategies – resolution, stalling, disclosure/concealment, and consultation – for managing these challenges.

Research, however, is needed to study whether these strategies will indeed enhance healthcare professional-patient communication. Studies can also focus on developing strategies for properly adjusting current medical ethics frameworks to address concrete social contexts, cultural milieu, religious beliefs, and other essential values that can complicate how information is managed within the clinical context. Notwithstanding these recommendations, health professionals should know that there are strategies they can adopt to address a variety of ethical challenges regarding information within the clinical context.

## Ethics approval and consent to participate

This study is a systematic review of the literature and does not involve human participants. No ethical approval was sought for this study.

## Notes

1 For example, the Australian Good Medical Practice: Code of Conduct for Doctors in Australia (2004: 9ff); the Council of International Organization of Medical Sciences” (CIOMS) “International Ethical Guidelines for Biomedical Research Involving Human Subjects” (2002, Commentaries on Guidelines 5, 16, 18 & 21) and “International Ethical Guidelines for Epidemiological Research (2009, Commentaries on Guidelines 4, 5, 21); the World Health Organization’s (WHO) “Draft Guidelines for Adverse Event Reporting and Learning System” (2005: 12ff) and “Ethical issues in Patient Safety Research” (2013: 30ff); the American Medical Association's (AMA) Code of Medical Ethics (Council on Ethical and Judicial Affairs, 2001:1ff); the World Medical Association's (WMA) International Code of Medical Ethics (1995); the Nigerian Code of Medical Ethics (2004); and the South African Guidelines for Good Practice in the Healthcare Professions (2008, Booklet 3: 2; Booklet 9: 4), to mention a few.

2 Disclosure is medically contraindicated if it would cause a depressed patient, for example, to become actively suicidal, or if it would compromise a patient's recovery process, for example, telling a hypertensive patient receiving critical care in the Intensive Care Unit that their spouse has just died.The American Medical Association's (AMA) Code of Medical Ethics (2010), for example, counsels that physicians have a “sacred duty … to avoid all things which have a tendency to discourage a patient's recovery and depress his spirits.” Disclosing news of the death of a loved one to a patient recieving care in an intensive care unit is therefore medically contraindicated if such news may hasten the death of patient or otherwise harm them.

3 We have previously published a separate study focusing on theoretical studies. See xxx.

4 See Also Klitzman & Weiss, 2006.

5 For example, more than half of respondents in one empirical study (Chaiyamahapurk et al., 2011), claim that they do not have enough time to provide required preventive and disclosure counselling for people living with HIV and AIDS. When patients refuse to disclose their sero-conversion to their partners, a majority of these health professionals kept the seroconversion confidential from the partner. Chaiyamahapurk et al., 2011.

6 The current practice in China is that family members receive bad news first. They then decide whether the patient should be informed or not.

7 This study defines communication broadly to include the content of communication, necessary communicative skills, and the judgment required for determining when communication is appropriate.

## References

[CIT0003] Akpinar, A., & Ersoy, N. (2014). Attitudes of physicians and patients towards disclosure of genetic information to spouse and first-degree relatives: A case study from Turkey. *BMC Medical Ethics*, *15*(1), 39. 10.1186/1472-6939-15-39 24885495 PMC4029893

[CIT0004] Alliman, S., Veach, P. M., Bartels, D. M., Lian, F., James, C., & Leroy, B. S. (2009). A comparative analysis of ethical and professional challenges experienced by Australian and U.S. genetic counselors. *Journal of Genetic Counseling*, *18*(4), 379–394. 10.1007/s10897-009-9229-9 19452265

[CIT0005] Andorno, R. (2004). The right not to know: An autonomy based approach. *Journal of Medical Ethics*, *30*(5), 435–439. 10.1136/jme.2002.001578 15467071 PMC1733927

[CIT0006] Beauchamp, T. L., & Childress, J. F. (2009). *Principles of biomedical ethics*. Oxford University Press.

[CIT0007] Beck, S., Van De Loo, A., & Reiter-Theil, S. (2008). A “little bit illegal"? Withholding and withdrawing of mechanical ventilation in the eyes of German intensive care physicians. *Medicine, Health Care and Philosophy*, *11*(1), 7–16. 10.1007/s11019-007-9097-8 17939061

[CIT0008] Bower, M. A., Veach, P. M., Bartels, D. M., & Leroy, B. S. (2002). A survey of genetic counselors’ strategies for addressing ethical and professional challenges in practice. *Journal of Genetic Counseling*, *11*(3), 163–186. 10.1023/A:1015275022199 12735294

[CIT0009] Buken, N. O., & Balseven-Odabasi, A. (2013). Physicians’ attitudes at the end-of-life: A cross-cultural evaluation. *Medicine and Law*, *32*, 549–565. 24552115

[CIT0010] Cahana, A., Weibel, H., & Hurst, S. A. (2008). Ethical decision-making: Do anesthesiologists, surgeons, nurse anesthetists, and surgical nurses reason similarly? *Pain Medicine*, *9*(6), 728–736. 10.1111/j.1526-4637.2007.00346.x 24640000

[CIT0011] Camoin, A., Dany, L., Tardieu, C., Ruquet, M., & Le Coz, P. (2018). Ethical issues and dentists’ practices with children with intellectual disability: A qualitative inquiry into a local French health network. *Disability and Health Journal*, *11*(3), 412–419. 10.1016/j.dhjo.2018.01.001 29396272

[CIT0012] Cantini, F., & Ells, C. (2007). The role of the clinical trial nurse in the informed consent process. *Canadian Journal of Nursing Research Archive*, *39*, 126–144. 17679589

[CIT0013] Chaiyamahapurk, S., Pannarunothai, S., & Nopkesorn, T. (2011). HIV prevention with positives and disclosure of HIV status: Practice and views of Thai healthcare providers. *Journal of the Medical Association of Thailand= Chotmaihet Thangphaet*, *94*(11), 1314–1320. 22256470

[CIT0014] Chih, A.-H., Su, P., Hu, W.-Y., Yao, C.-A., Cheng, S.-Y., Lin, Y.-C., & Chiu, T.-Y. (2016). The changes of ethical dilemmas in palliative care A lesson learned from comparison between 1998 and 2013 in Taiwan. *Medicine*, *95*(1), e2323. 10.1097/MD.0000000000002323 26735533 PMC4706253

[CIT0015] Chiu, T. Y., Hu, W. Y., Huang, H. L., Yao, C. A., & Chen, C. Y. (2009). Prevailing ethical dilemmas in terminal care for patients with cancer in Taiwan. *Journal of Clinical Oncology*, *27*(24), 3964–3968. 10.1200/JCO.2008.21.4643 19470919

[CIT0016] Duval, G., Clarridge, B., Gensler, G., & Danis, M. (2004). A national survey of U.S. Internists’ experiences with ethical dilemmas and ethics consultation. *Journal of General Internal Medicine*, *19*(3), 251–258. 10.1111/j.1525-1497.2004.21238.x 15009780 PMC1492156

[CIT0017] Elger, B. S., Handtke, V., & Wangmo, T. (2015). Informing patients about limits to confidentiality: A qualitative study in prisons. *International Journal of Law and Psychiatry*, *41*, 50–57. 10.1016/j.ijlp.2015.03.007 25862306

[CIT0018] Elwyn, T. S., Fetters, M. D., Sasaki, H., & Tsuda, T. (2002). Responsibility and cancer disclosure in Japan. *Social Science &amp; Medicine*, *54*(2), 281–293. 10.1016/S0277-9536(01)00028-4 11824932

[CIT0019] Epstein, E. G., & Hamric, A. B. (2009). Moral distress, moral residue, and the crescendo effect. *Journal of Clinical Ethics*, *20*(4), 330–342. 20120853

[CIT0020] Erde, E. L., Mccormack, M. K., Steer, R. A., Ciervo, C. A., & Mcabee, J. R., & N, G. (2006). Patient confidentiality vs disclosure of inheritable risk: A survey-based study. *Journal of the American Osteopathic Association*, *106*(10), 615–620. 17122032

[CIT0021] Ewuoso, O. C., Hall, S., & Dierickx, K. (2017). How do healthcare professionals manage ethical challenges regarding information in healthcare professional-patient clinical interactions? A review of concept/argument-based papers and case analyses. *South African Journal of Bioethics and Law*, *10*(2), 75–82.

[CIT0022] Falk, M. J., Dugan, R. B., O'Riordan, M. A., Matthews, A. L., & Robin, N. H. (2003). Medical geneticists’ duty to warn at-risk relatives for genetic disease. *American Journal of Medical Genetics*, *120A*(3), 374–380. 10.1002/ajmg.a.20227 12838558

[CIT0023] Fennig, S., Secker, A., Levkovitz, Y., Barak, V., Benyakar, M., Farina, J., Roe, D., Treves, I., & Fennig, S. (2004). Are psychotherapists consistent in their ethical attitude to patient confidentiality? *The Israel Journal of Psychiatry and Related Sciences*, *41*(2), 82–89. 15478453

[CIT0024] George, E., & Demir Kureci, H. (2020). Attitudes towards and experiences of ethical dilemmas in treatment decision-making process among medical oncologists. *International Journal of Clinical Pharmacy*, *26*(1), 209–215. 10.1111/jep.13127 30912249

[CIT0025] Godfrey, G., Hilton, A., & Bellomo, R. (2013). To treat or not to treat: Withholding treatment in the ICU. *Current Opinion in Critical Care*, *19*(6), 624–629. 10.1097/MCC.0000000000000036 24240829

[CIT0027] Groepper, D., Mccarthy Veach, P., Leroy, B. S., & Bower, M. (2015). Ethical and professional challenges encountered by laboratory genetic counselors. *Journal of Genetic Counseling*, *24*(4), 580–596. 10.1007/s10897-014-9787-3 25398381

[CIT0028] Gronlund, C. F., Dahlqvist, V., Zingmark, K., Sandlund, M., & Soderberg, A. (2016). Managing ethical difficulties in healthcare: Communicating in inter-professional Clinical Ethics support sessions. *HEC Forum*, *28*(4), 321–338. 10.1007/s10730-016-9303-2 27147521

[CIT0029] Halvorsen, K., Slettebo, A., Nortvedt, P., Pedersen, R., Kirkevold, M., Nordhaug, M., & Brinchmann, B. S. (2008). Priority dilemmas in dialysis: The impact of old age. *Journal of Medical Ethics*, *34*(8), 585–589. 10.1136/jme.2007.022061 18667645

[CIT0030] Hattingh, H. L., & King, M. A. (2019). Pharmacy ethical reasoning: A comparison of Australian pharmacists and interns. *International Journal of Clinical Pharmacy*, *41*(4), 1085–1098. 10.1007/s11096-019-00815-5 31093939

[CIT0031] Hawker, S., Payne, S., Kerr, C., Hardey, M., & Powell, J. (2002). Appraising the evidence: Reviewing disparate data systematically. *Qualitative Health Research*, *12*(9), 1284–1299. 10.1177/1049732302238251 12448672

[CIT0032] Helft, P. R., Chamness, A., Terry, C., & Uhrich, M. (2011). Oncology nurses’ attitudes toward prognosis-related communication: A pilot mailed survey of oncology nursing society members. *Oncology Nursing Forum*, *38*(4), 468–474. 10.1188/11.ONF.468-474 21708537

[CIT0033] Huijer, M., Van Leeuwen, E., Boenink, A., & Kimsma, G. (2000). Medical students' cases as an empirical basis for teaching clinical ethics. *Academic Medicine*, *75*(8), 834–839. 10.1097/00001888-200008000-00017 10965863

[CIT0034] Hurst, S. A., Perrier, A., Pegoraro, R., Reiter-Theil, S., Forde, R., Slowther, A.-M., Garrett-Mayer, E., & Danis, M. (2007). Ethical difficulties in clinical practice: Experiences of European doctors. *Journal of Medical Ethics*, *33*(1), 51–57. 10.1136/jme.2005.014266 17209113 PMC2598078

[CIT0034a] International Labour Organization. (2012). International Standard Classification of Occupations. Geneva: ILO, 2012. http://www.ilo.org/wcmsp5/groups/public/---dgreports/---dcomm/---publ/documents/publication/wcms_172572.pdf

[CIT0035] Jegede, A. S. (2009). African ethics, Health Care Research and community and individual participation. *Journal of Asian and African Studies*, *44*(2), 239–253. 10.1177/0021909608101412

[CIT0036] Joseph, J. W., Marshall, K. D., Reich, B. E., Boyle, K. L., Hill, K. P., Weiner, S. G., & Derse, A. R. (2019). How emergency physicians approach refusal of observation after naloxone resuscitation. *PLoS One*. 10.1016/j.jemermed.2019.09.021 31753755

[CIT0037] Jurchak, M., Grace, P. J., Lee, S. M., Willis, D. G., Zollfrank, A. A., & Robinson, E. M. (2017). Developing abilities to navigate through the grey zones in complex environments: Nurses’ reasons for applying to a clinical ethics residency for nurses. *Journal of Nursing Scholarship*, *49*(4), 445–455. 10.1111/jnu.12297 28605124

[CIT0038] Kadivar, M., Mardani-Hamooleh, M., & Shayestefar, S. (2017). Evaluation of pediatric residents’ attitudes toward ethical conflict: A cross-sectional study in Tehran, Iran. *Journal of Medical Ethics and History of Medicine*, *10*, 2. 28523117 PMC5432945

[CIT0039] Kagan, I., Ovadia, K. L., & Kaneti, T. (2008). Physicians’ and nurses’ views on infected healthcare workers. *Nursing Ethics*, *15*(5), 573–585. 10.1177/0969733007088362 18687813

[CIT0040] Kebede, B. G., Abraha, A., Andersson, R., Munthe, C., Linderholm, M., Linderholm, B., & Berbyuk Lindström, N. (2020). Communicative challenges among physicians, patients, and family caregivers in cancer care: An exploratory qualitative study in Ethiopia. *PloS one*, *15*, e0230309. 10.1371/journal.pone.0230309 32168353 PMC7069641

[CIT0041] Klitzman, R., & Weiss, J. (2006). Disclosures of illness by doctors to their patients: A qualitative study of doctors with HIV and other serious disorders. *Patient Education and Counseling*, *64*(1-3), 277–284. 10.1016/j.pec.2006.03.006 16860516

[CIT0042] Krautscheid, L., & Brown, M. (2014). Microethical decision-making among baccalaureate nursing students: A qualitative investigation. *Journal of Nursing Education.*, *53*, S19–S25. 10.3928/01484834-20140211-05 24512334

[CIT0043] Kvist, T., Wickstrom, A., Miglis, I., & Dahllof, G. (2014). The dilemma of reporting suspicions of child maltreatment in pediatric dentistry. *European Journal of Oral Sciences*, *122*(5), 332–338. 10.1111/eos.12143 25039643

[CIT0044] Kwon, J. H., Baek, S. K., Kim, B. S., Koh, S. J., Ahn, H. K., Lim, J. H., Lim, C., & Kim, D. Y. (2019). Surrogate decision-making of chemotherapy consent: Do we really provide informed consent of chemotherapy for patients? *The Korean Journal of Internal Medicine*, *34*(3), 626–633. 10.3904/kjim.2017.252 29843493 PMC6506731

[CIT0045] Lapid, M., Moutier, C., Dunn, L., Hammond, K. G., & Roberts, L. W. (2009). Professionalism and ethics education on relationships and boundaries: Psychiatric residents’ training preferences. *Academic Psychiatry*, *33*(6), 461–469. 10.1176/appi.ap.33.6.461 19933889

[CIT0046] Laurie, G. (2014). Recognizing the right not to know: Conceptual, professional, and legal implications. *Journal of Law, Medicine &amp; Ethics*, *42*(1), 53–63. 10.1111/jlme.12118 26767476

[CIT0047] Lisker, R., & Carnevale, A. (2006). Changing opinions of Mexican geneticists on ethical issues. *Archives of Medical Research*, *37*(6), 794–803. 10.1016/j.arcmed.2006.01.005 16824941

[CIT0048] Lokker, M. E., Swart, S. J., Rietjens, J. A. C., Van Zuylen, L., Perez, R., & Van Der Heide, A. (2018). Palliative sedation and moral distress: A qualitative study of nurses. *Applied Nursing Research*, *40*, 157–161. 10.1016/j.apnr.2018.02.002 29579492

[CIT0049] Lotz, J. D., Jox, R. J., Meurer, C., Borasio, G. D., & Fuhrer, M. (2016). Medical indication regarding life-sustaining treatment for children: Focus groups with clinicians. *Palliative Medicine*, *30*(10), 960–970. 10.1177/0269216316628422 26847523 PMC5117124

[CIT0051] Lützén, K., Johansson, A., & Nordström, G. (2000). Moral sensitivity: Some differences between nurses and physicians. *Nursing Ethics*, *7*(6), 520–530. 10.1177/096973300000700607 11221393

[CIT0052] Macdonald, S. I., & Shemie, S. D. (2017). Ethical challenges and the donation physician specialist: A scoping review. *Transplantation*, *101*(5S), S27–s40. 10.1097/TP.0000000000001697 28437369

[CIT0053] Malcolm, D., & Scott, A. (2014). Practical responses to confidentiality dilemmas in elite sport medicine. *British Journal of Sports Medicine*, *48*(19), 1410–1413. 10.1136/bjsports-2013-092458 23881893

[CIT0054] Martin, B. M., Love, T. P., Srinivasan, J., Sharma, J., Pettitt, B., Sullivan, C., Pattaras, J., Master, V. A., & Brewster, L. P. (2014). Designing an ethics curriculum to support global health experiences in surgery. *Journal of Surgical Research*, *187*(2), 367–370. 10.1016/j.jss.2013.06.013 24472281

[CIT0055] Mccahill, L. E., Krouse, R. S., Chu, D. Z., Juarez, G., Uman, G. C., Ferrell, B. R., & Wagman, L. D. (2002). Decision-making in palliative surgery. *Journal of the American College of Surgeons*, *195*(3), 411–422.; discussion 422–3. 10.1016/S1072-7515(02)01306-6 12229950

[CIT0056] Molina-Mula, J., Gallo-Estrada, J., & Perello-Campaner, C. (2017). Impact of interprofessional relationships from nurses’ perspective on the decision-making capacity of patients in a clinical setting. *International Journal of Environmental Research and Public Health*, *15*(1). 10.3390/ijerph15010049 PMC5800148 29286342

[CIT0057] Morparia, K., Dickerman, M., & Hoehn, K. S. (2012). Futility: Unilateral decision-making is not the default for pediatric intensivists. *Pediatric Critical Care Medicine*, *13*(5), e311–e315. 10.1097/PCC.0b013e31824ea12c 22760427

[CIT0058] Murray, B. (2012). Informed consent: What must a physician disclose to a patient? *AMA Journal of Ethics*, *14*(7), 563–566. 10.1001/virtualmentor.2012.14.7.hlaw1-1207 23351294

[CIT0059] Nash, M. J., & Romanos, M. T. (2010). An exploration of mental health nursing students’ experiences and attitudes towards using cigarettes to change client's behaviour. *Journal of Psychiatric and Mental Health Nursing*, *17*(8), 683–691. 10.1111/j.1365-2850.2010.01605.x 21050334

[CIT0060] Nordam, A., Torjuul, K., & Sorlie, V. (2005). Ethical challenges in the care of older people and risk of being burned out among male nurses. *Journal of Clinical Nursing*, *14*(10), 1248–1256. 10.1111/j.1365-2702.2005.01230.x 16238771

[CIT0061] Odeniyi, F., Nathanson, P. G., Schall, T. E., & Walter, J. K. (2017). Communication challenges of oncologists and intensivists caring for pediatric oncology patients: A qualitative study. *Journal of Pain and Symptom Management*, *54*(6), 909–915. 10.1016/j.jpainsymman.2017.06.013 28807699

[CIT0062] Palmboom, G. G., Willems, D. L., Janssen, N. B., & De Haes, J. C. (2007). Doctor's views on disclosing or withholding information on low risks of complication. *Journal of Medical Ethics*, *33*(2), 67–70. 10.1136/jme.2005.014936 17264190 PMC2598232

[CIT0063] Pettersson, M., Hedström, M., & Höglund, A. T. (2018). Ethical competence in DNR decisions -a qualitative study of Swedish physicians and nurses working in hematology and oncology care. *BMC Medical Ethics*, *19*(1), 63. 10.1186/s12910-018-0300-7 29914440 PMC6007064

[CIT0064] Pickles, K., Carter, S. M., Rychetnik, L., Mccaffery, K., & Entwistle, V. A. (2016). General practitioners’ experiences of, and responses to, uncertainty in prostate cancer screening: Insights from a qualitative study. *PLoS One*, *11*(4), e0153299. 10.1371/journal.pone.0153299 27100402 PMC4839572

[CIT0065] Pillastrini, P., Vanti, C., Curti, S., Mattioli, S., Ferrari, S., Violante, F. S., & Guccione, A. (2015). Using PubMed search strings for efficient retrieval of manual therapy Research literature. *Journal of Manipulative and Physiological Therapeutics*, *38*(2), 159–166. 10.1016/j.jmpt.2014.11.005 25499192

[CIT0066] Prentice, T., Janvier, A., Gillam, L., & Davis, P. G. (2016). Moral distress within neonatal and paediatric intensive care units: A systematic review. *Archives of Disease in Childhood*, *101*(8), 701–708. 10.1136/archdischild-2015-309410 26801075

[CIT0067] Pye, K. (2013). Exploring moral distress in pediatric oncology; a sample of registered practitioners. *Issues in Comprehensive Pediatric Nursing*, *36*(4), 248–261. 10.3109/01460862.2013.812693 24001179

[CIT0068] Roberts, J. K., Hargett, C. W., Nagler, A., Jakoi, E., & Lehrich, R. W. (2015). Exploring student preferences with a Q-sort: The development of an individualized renal physiology curriculum. *Advances in Physiology Education*, *39*(3), 149–157. 10.1152/advan.00028.2015 26330030 PMC4747902

[CIT0069] Ruhnke, G. W., Wilson, S. R., Akamatsu, T., Kinoue, T., Takashima, Y., Goldstein, M. K., Koenig, B. A., Hornberger, J. C., & Raffin, T. A. (2000). Ethical decision-making and patient autonomy: A comparison of physicians and patients in Japan and the United States. *Chest*, *118*(4), 1172–1182. 10.1378/chest.118.4.1172 11035693

[CIT0070] Schimmer, C., Gorski, A., Ozkur, M., Sommer, S. P., Hamouda, K., Hain, J., Aleksic, I., & Leyh, R. (2012). Policies of withholding and withdrawal of life-sustaining treatment in critically ill patients on cardiac intensive care units in Germany: A national survey. *Interactive CardioVascular and Thoracic Surgery*, *14*(3), 294–299. 10.1093/icvts/ivr119 22194277 PMC3290372

[CIT0071] Sørlie, V., Lindseth, A., Udén, G., & Norberg, A. (2000). Women Physicians’ narratives about being in ethically difficult care situations in paediatrics. *Nursing Ethics*, *7*(1), 47–62. 10.1177/096973300000700107 10703423

[CIT0072] Span-Sluyter, C., Lavrijsen, J. C. M., Van Leeuwen, E., & Koopmans, R. (2018). Moral dilemmas and conflicts concerning patients in a vegetative state/unresponsive wakefulness syndrome: Shared or non-shared decision-making? A qualitative study of the professional perspective in two moral case deliberations. *BMC Medical Ethics*, *19*(1), 10. 10.1186/s12910-018-0247-8 29471814 PMC5824545

[CIT0073] Swaminath, G. (2008). The doctor's dilemma: Truth telling. *Indian Journal of Psychiatry*, *50*(2), 83–84. 10.4103/0019-5545.42392 19742214 PMC2738333

[CIT0074] Swetz, K. M., Crowley, M. E., Hook, C. C., & Mueller, P. S. (2007). Report of 255 clinical ethics consultations and review of the literature. *Mayo Clinic Proceedings*, *82*(6), 686–691. 10.1016/S0025-6196(11)61188-9 17550748

[CIT0075] Thomas, T. A., & Mccullough, L. B. (2015). A philosophical taxonomy of ethically significant moral distress. *Journal of Medicine and Philosophy*, *40*(1), 102–120. 10.1093/jmp/jhu048 25503608

[CIT0076] Torjuul, K., Nordam, A., & Sorlie, V. (2005b). Ethical challenges in surgery as narrated by practicing surgeons. *BMC Medical Ethics*, *6*(1), E2. 10.1186/1472-6939-6-2 15737235 PMC553983

[CIT0077] Vaga, B. B., Moland, K. M., & Blystad, A. (2016). Boundaries of confidentiality in nursing care for mother and child in HIV programmes. *Nursing Ethics*, *23*(5), 576–586. 10.1177/0969733015576358 25956154

[CIT0078] Van Zuuren, F. J., & Van Manen, E. (2006). Moral dilemmas in neonatology as experienced by healthcare practitioners: A qualitative approach. *Medicine, Health Care and Philosophy*, *9*(3), 339–347. 10.1007/s11019-005-5641-6 17082871

[CIT0079] Velan, B. (2019). Truth-telling and doctor-assisted death as perceived by Israeli physicians. *Journal of Evaluation in Clinical Practice*, *20*, 13. 10.1186/s12910-019-0350-5 PMC6380017 30777058

[CIT0080] Watermeyer, J. (2015). ‘Are we allowed to disclose?': A healthcare team's experiences of talking with children and adolescents about their HIV status. *Health Expectations*, *18*(4), 590–600. 10.1111/hex.12141 24112299 PMC5060803

[CIT0081] Watts, S., & Stenner, P. (2005). Doing Q methodology: Theory, method and interpretation. *Qualitative Research in Psychology*, *2*(1), 67–91. 10.1191/1478088705qp022oa

[CIT0082] Westra, A. E., Willems, D. L., & Smit, B. J. (2009). Communicating with Muslim parents: “the four principles” are not as culturally neutral as suggested. *European Journal of Pediatrics*, *168*(11), 1383–1387. 10.1007/s00431-009-0970-8 19306021 PMC2745533

[CIT0083] Williams, C., Alderson, P., & Farsides, B. (2002). Dilemmas encountered by health practitioners offering nuchal translucency screening: A qualitative case study. *Prenatal Diagnosis*, *22*(3), 216–220. 10.1002/pd.289 11920897

[CIT0084] World Medical Association. (1995). The international code of medical ethics. *Medical Ethics*, *3*(4), 78. 15586945

[CIT0085] Wuensch, A., Tang, L., Goelz, T., Zhang, Y., Stubenrauch, S., Song, L., Hong, Y., Zhang, H., Wirsching, M., & Fritzsche, K. (2013). Breaking bad news in China–the dilemma of patients’ autonomy and traditional norms. A first communication skills training for Chinese oncologists and caretakers. *Psycho-oncology*, *22*(5), 1192–1195. 10.1002/pon.3112 22639333

[CIT0086] Yang, C. F., Che, H. L., Hsieh, H. W., & Wu, S. M. (2016). Concealing emotions: Nurses’ experiences with induced abortion care. *Journal of Clinical Nursing*, *25*(9-10), 1444–1454. 10.1111/jocn.13157 26991834

[CIT0087] Yildiz, E. (2019). Ethics in nursing: A systematic review of the framework of evidence perspective. *Nursing Ethics*, *26*(4), 1128–1148. 10.1177/0969733017734412 29166840

[CIT0088] Yoon, J. D., Rasinski, K. A., & Curlin, F. A. (2010). Conflict and emotional exhaustion in obstetrician-gynaecologists: A nationDDdDal survey. *Journal of Medical Ethics*, *36*(12), 731–735. 10.1136/jme.2010.037762 21112936 PMC3632257

